# 

**Published:** 1845-09

**Authors:** 


Page

### LIBRARY.

Anatomy of the Dental System, Human and Comparative, Translated from the French of Ph. Fr. Blandin, Surgeon of the Hotel Dieu, &c., by Robert Arthur, D. D. S., ........ 17

### JOURNAL.

ARTICLE I.—Address delivered before the American Society of Dental Surgeons. By Eleazar Parmly, M. D., ........ 3
ARTICLE II.—Dissertation on Practical Dentistry. By E. J. Dunning. 15
ARTICLE III.—Dissertation on the Elevation of the Dental Profession. By E. Townsand, D. D. S., ........ 25
ARTICLE IV.—Address on the Pursuit of Professional Excellence. By John Allen, D. D. S., ........ 30
ARTICLE V.—Dissertation on the State of the Dental Profession, and Dental Empiricism. By James Taylor, D. D. S., ........ 38
ARTICLE VI.—Is the Negro subject to Hare-lip? ........ 58
ARTICLE VII.—Letter from E. S. Bennett, M. D., ........ 60
ARTICLE VIII.—General Items suggested by a Review of the Minutes of the Last Meeting. By the Syracuse Editor, ........ 61

### BIBLIOGRAPHICAL NOTICES.

Observations on the Growth and Irregularities of Children's Teeth. By W. H. Mortimer, late Surgeon Dentist to the British Embassy at Paris, 69
Medicines, their Use and Mode of Administration. By J. Moore Neligan, M. D., Physician to Lewis street Hospital, and Lecturer on Materia Medica and Therapeutics in the Dublin School of Medicine, ........ 70
The Half-Yearly Abstract of the Medical Sciences. Edited by W. H. Ranking, M. D., Cantab. Physician to the Suffolk General Hospital, 71
Medical Lexicon, or Modern Terminology. By David Meredith Reese, A. M., M. D., late Professor of the Institutes of Medicine and Surgery, and Medical Jurisprudence, in the Washington University of Baltimore, &c. ........ 72
Stockton's Dental Intelligencer, ........ 72
The British American Journal of Medical and Physical Science, ........ 73
The Buffalo Medical Journal, ........ 73

### MISCELLANEOUS NOTICES.

Sixth Annual Meeting of the American Society of Dental Surgeons, ........ 74
The Proceedings of the American Society of Dental Surgeons, ........ 86
The American Journal and Library of Dental Science, ........ 86
Washington City Editor, ........ 87
Removal of a Drill-head from the Cavity of a Tooth, by means of a Magnet, 87
To Subscribers in Great Britain, ........ 88
Improved Drill Stock, ........ 88
Dental Infirmary, ........ 88
To Correspondents, ........

